# Survival, by Birth Weight and Gestational Age, in Individuals With Congenital Heart Disease: A Population‐Based Study

**DOI:** 10.1161/JAHA.116.005213

**Published:** 2017-07-21

**Authors:** Kate E. Best, Peter W.G. Tennant, Judith Rankin

**Affiliations:** ^1^ Institute of Health & Society Newcastle University Newcastle upon Tyne United Kingdom; ^2^ School of Healthcare University of Leeds Leeds United Kingdom

**Keywords:** congenital, congenital cardiac defect, survival, survival analysis, Risk Factors, Epidemiology, Mortality/Survival

## Abstract

**Background:**

Congenital heart disease (CHD) survival estimates are important to understand prognosis and evaluate health and social care needs. Few studies have reported CHD survival estimates according to maternal and fetal characteristics. This study aimed to identify predictors of CHD survival and report conditional survival estimates.

**Methods and Results:**

Cases of CHD (n=5070) born during 1985–2003 and notified to the Northern Congenital Abnormality Survey (NorCAS) were matched to national mortality information in 2008. Royston–Parmar regression was performed to identify predictors of survival. Five‐year survival estimates conditional on gestational age at delivery, birth weight, and year of birth were produced for isolated CHD (ie, CHD without extracardiac anomalies). Year of birth, gestational age, birth weight, and extracardiac anomalies were independently associated with mortality (all *P*≤0.001). Five‐year survival for children born at term (37–41 weeks) in 2003 with average birth weight (within 1 SD of the mean) was 96.3% (95% CI, 95.6–97.0). Survival was most optimistic for high‐birth‐weight children (>1 SD from the mean) born post‐term (≥42 weeks; 97.9%; 95% CI, 96.8–99.1%) and least optimistic for very preterm (<32 weeks) low‐birth‐weight (<1 SD from mean) children (78.8%; 95% CI, 72.8–99.1).

**Conclusions:**

Five‐year CHD survival is highly influenced by gestational age and birth weight. For prenatal counseling, conditional survival estimates provide best‐ and worst‐case scenarios, depending on final gestational age and birth weight. For postnatal diagnoses, they can provide parents with more‐accurate predictions based on their baby's birth weight and gestational age.

## Introduction

Congenital heart disease (CHD) is a range of structural anomalies of the heart, which affect around 1% of births.^1^ Individuals with CHD often require complex life‐saving surgeries in infancy and lifetime follow‐up.^2^ Survival estimates are therefore important to understand prognosis and evaluate health and social care needs. Our recent systematic review of 16 population‐based studies estimated a 5‐year survival of 85%, ranging from 14% for hypoplastic left heart to 96% for ventricular septal defect.^3^ CHD survival is strongly influenced by gestational age at birth, birth weight, and the presence of extracardiac anomalies (ie, additional congenital anomalies outside the cardiovascular system),^4^, ^5^, ^6^, ^7^, ^8^, ^9^ but there is conflicting evidence for plurality, maternal age, ethnicity, and socioeconomic deprivation.^4^, ^5^, ^6^, ^7^, ^8^, ^9^, ^10^, ^11^ Previous studies of CHD mortality tend to report hazard ratios (HRs) without reporting survival estimates, which have greater utility for counseling and service planning.

The aim of this study is to estimate long‐term survival of individuals with CHD, born in the North of England during 1985–2003, conditioned on important determinants.

## Methods

### Case Inclusion

The Northern Congenital Abnormality Survey (NorCAS) is a population‐based register that collects data on cases of congenital anomalies delivered to women residing in the North of England (Figure). Cases diagnosed up to age 16 years (age 12 years after 2001) are notified from multiple sources, including prenatal ultrasound, fetal medicine, cytogenetic laboratories, the regional cardiology center, pathology and paediatric surgery, and coded using the World Health Organization International Classification of Diseases.

**Figure 1:**
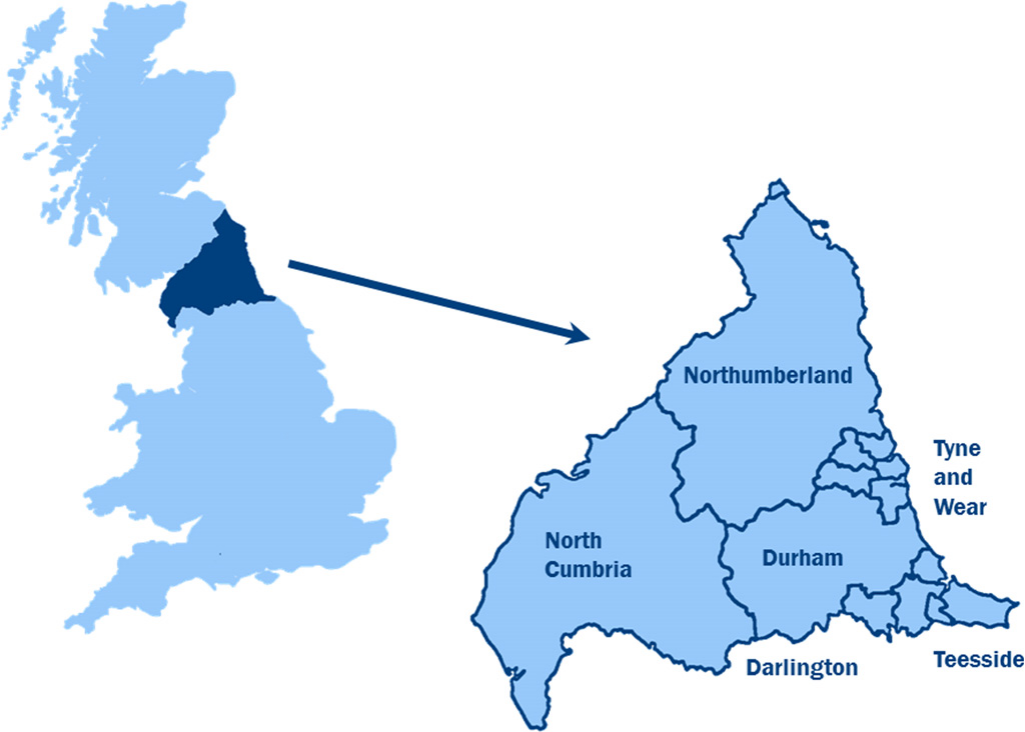
Map showing the area covered by the Northern Congenital Abnormality Survey.

Cases with CHD (International Classification of Diseases, Tenth Revision: Q20–26) live born between January 1, 1985 and December 31, 2003 were included. Cases with a single minor CHD, for example, patent ductus arteriosus born <37 weeks' gestation, were excluded as per European Surveillance of Congenital Anomalies guidelines.^12^

### Survival Status

Deaths before age 1 year were identified from the region's Perinatal Mortality Survey, which is linked to the NorCAS through the mother's details. The Perinatal Mortality Survey collects data on infant deaths from statutory death registrations for infants whose mothers reside in the North of England (Figure). Deaths beyond age 1 year were identified by linking with national death registrations from the Office for National Statistics. The linkage was performed on January 28, 2008 using “fuzzy matching” by infant name, last known address, mother's age at delivery and sex. Cases not on the Perinatal Mortality Survey or matched with Office for National Statistics records (eg, birth certificates) were cross‐referenced with the NorCAS or hospital records and located through the National Health Service National Strategic Tracing Service. Those that could not be traced were excluded (0.5%). Cases with matched death registrations were classified as deceased on their date of death; those without were coded as alive and censored on the date the linkage was performed.

### Ethical Approval

The NorCAS has approval from the Confidentiality Advisory Group of the Health Research Authority (PIAG 2‐08(e)/2002) to hold data without consent and, at the time of study, ethics approval (09/H0405/48) to undertake research involving the data. This study was given a favorable ethical opinion by the South Tees Local Research Ethics Committee.

### Data

Using a fetal growth formula, birth weight at 40 weeks gestational age was predicted for all cases (based on their actual birth weight and gestational age).^13^ Regional birth weight references were applied to this formula, with standardization for gestational age at birth, sex, and plurality.^13^, ^14^

From mothers' residential postcode at delivery, the Index of Multiple Deprivation 2004 was calculated. Index of Multiple Deprivation is a measure of area‐level socioeconomic deprivation calculated from income, employment, health, education, access to services, social environment, housing stress, living environment, and crime.^15^, ^16^ Index of Multiple Deprivation rank, which ranges from 1 for the most deprived area to 32 844 for the least deprived area, was categorized as least, average, and most deprived tertiles. The tertiles were created regionally to allow almost equal numbers of cases to be assigned to each category.

The following were investigated as risk factors of mortality: year of birth (continuous); maternal age at birth (continuous); extracardiac anomalies (none, structural, or chromosomal/genetic); standardized birth weight (low [z <−1], average [−1≤ z ≤1], high [z >1]); gestational age at birth (very preterm [<32 weeks]; moderate preterm [32–36 weeks]; term [37–41 weeks]; post‐term [≥42 weeks]); socioeconomic deprivation (least, average, or most deprived), infant sex (male or female); and plurality (singleton or twin). CHD severity was coded into 4 groups (I [most severe], II, III [least severe], and “unclassified”), using a slightly adapted version of Khoshnood et al's coding system.^17^ Unclassified CHD includes patent ductus (≥37 weeks), congenital heart block, dextrocardia, and aortic regurgitation, among others. Cases with multiple subtypes were coded according to the most severe and thus may vary from those previously reported from these data.^18^ Table 1 shows which CHD subtypes are in each severity category.

**Table 1:** Survival for All CHD According to Risk Factors

Risk Factors	N (%)	HR (95% CI)	*P* Value	aHR (95% CI)	*P* Value
Year of birth (per y)	5070 (100)	0.93 (0.91–0.94)	<0.001	0.94 (0.92–0.95)	<0.001
Gestational age					
Very preterm (<32 weeks)	149 (2.9)	4.33 (3.3–5.67)	<0.001	6.85 (5.11–9.18)	<0.001
Moderately preterm (32–36 weeks)	619 (12.2)	2.09 (1.74–2.53)		1.87 (1.54–2.27)	
Term (37–41 weeks)	4040 (79.7)	1 (reference category)		1 (reference category)	
Post‐term (≥42 weeks)	172 (3.4)	0.73 (0.43–1.21)		0.65 (0.38–1.08)	
Missing	90 (1.8)				
Birth weight					
Low (z <−1)	1274 (59.0)	1.73 (1.48–2.03)	<0.001	1.31 (1.11–1.55)	0.001
Average (−1≤ z ≤1)	2935 (25.6)	1 (reference category)		1 (reference category)	
High (z >1)	770 (15.5)	0.83 (0.65–1.06)		0.76 (0.59–0.98)	
Missing	91				
Extracardiac anomalies					
None	4181 (82.5)	1 (reference category)	<0.001	1 (reference category)	<0.001
Structural	287 (5.7)	4.22 (3.38–5.27)		2.95 (2.34–3.72)	
Chromosomal/genetic	602 (11.9)	4.11 (3.47–4.87)		3.07 (2.57–3.67)	
Maternal age (per y)	4795 (94.6)	1.01 (1.00–1.02)	0.04	1.01 (1.00–1.02)	0.10
Missing	275 (5.4)				
Sex					
Male	2675 (52.8)	1 (reference category)	0.65	1 (reference category)	0.64
Female	2395 (47.2)	0.96 (0.83–1.12)		1.05 (0.9–1.22)	
Deprivation					
Most deprived	1690 (33.3)	1 (reference category)	0.11	1 (reference category)	0.20
Average deprived	1690 (33.3)	0.94 (0.78–1.12)		0.93 (0.78–1.11)	
Least deprived	1689 (33.3)	0.82 (0.69–0.99)		0.84 (0.69–1.02)	
Missing	1 (0.02)				
Plurality					
Singleton	4918 (97.0)	1 (reference category)	0.009	1 (reference category)	0.22
Multiple	151 (3.0)	1.61 (1.13–2.31)		0.78 (0.53–1.15)	
Missing	1 (0.02)				
Severity			<0.001		
I (most)	163 (3.2)	4.26 (3.45–5.25)		5.73 (4.61–7.13)	
II	1579 (31.1)	1 (reference category)		1 (reference category)	
III (least)	3032 (59.8)	0.15 (0.12–0.18)		0.19 (0.15–0.23)	
Unclassified	296 (5.8)	0.55 (0.40–0.75)		0.49 (0.36–0.67)	

aHR indicates adjusted hazard ratio; CHD, **c**ongenital heart disease; HR, hazard ratio.

### Statistical Analysis

Unadjusted and adjusted HRs were estimated using proportional hazards Royston–Parmar models, with 1 knot. Parametric models such as Royston–Parmar models differ from the traditionally used semiparametric Cox model because they directly model the baseline hazard.^19^ This results in smoother and therefore more‐precise predictions of survival than those resulting from the Cox model. The Royston–Parmar approach in particular makes no specific distributional assumptions (as is the case for other parametric approaches such as the Weibull or exponential models), instead modeling and smoothing the baseline hazard using cubic splines to maximise model accuracy. Mean 5‐year survival estimates for composite CHD were estimated using a Royston–Parmar model adjusted for birth weight, gestational age, extracardiac anomalies, and year of birth, and reported for isolated CHD (ie, no extracardiac anomalies) during the study's most recent year of birth (ie, 2003). Where there were 5 or more deaths per variable, mean survival estimates were also estimated for individual CHD subtypes. Here, Royston–Parmar models were fitted with dichotomization of gestational age (preterm [<37 weeks] versus term [≥37 weeks]) and presence of extracardiac anomalies (none versus any), attributed to low numbers. Ninety‐five percent CIs were estimated using the Delta method.

The proportional hazards assumption was checked using log‐log plots and by comparing HRs for different categorizations of survival time. Martingale residuals were plotted against continuous variables to check that the functional form was linear. *P*<0.05 was considered statistically significant and analyses were performed in Stata software (version 14; StataCorp LP, College Station, TX).

## Results

Of 5093 cases of CHD, 5070 (99.5%) had known survival status. Of these, 87.1% (95% CI, 86.2–88.0) survived to age 5 years, 86.7% (95% CI, 85.7–87.6%) to age 10 years and 85.2% (95% CI, 84.1–86.3) to age 20 years. Of cases with isolated CHD, 91.2% (95% CI, 90.3–92.0) survived to age 5 years, 90.9% (95% CI, 90.0–91.8) to age 10 years, and 89.7% (95% CI, 88.5–90.7) to age 20 years.

### Predictors of Survival

Year of birth (*P*<0.001), gestational age at birth (*P*<0.001), birth weight (*P*<0.001), extracardiac anomalies (*P*<0.001), CHD severity (*P*<0.001), maternal age (*P*=0.04), and plurality of pregnancy (*P*=0.009) were all crudely associated with survival (Table 1). For each year increase in year of birth, the risk of mortality decreased by 7% (HR=0.93; 95% CI, 0.92–0.94). Cases born very preterm and moderately preterm were 4.33 (95% CI, 3.30–5.67; *P*<0.001) and 2.09 (95% CI, 1.74–2.52; *P*<0.001) times more likely to result in mortality than term cases. Cases born post‐term were 27% less likely to result in mortality (HR=0.73; 95% CI, 0.43–1.21), although this was not statistically significant (*P*=0.22). Compared with average birth‐weight cases, low birth weight cases were at increased risk of mortality (HR=1.73; 95% CI, 1.48–2.03; *P*<0.001) and high birth weight cases were at decreased risk of mortality, although this did not reach statistical significance in the univariable model (HR=0.83; 95% CI, 0.65–1.06; *P*=0.13). For each year increase in maternal age, the risk of mortality increased by 1% (HR=1.01; 95% CI, 1.00–1.02). The risk of mortality was 18% lower in cases resident in the region's third least deprived areas compared with the third most deprived (95% CI, 0.68–0.98; *P*=0.04), although the total effect of socioeconomic deprivation was not statistically significant (*P*=0.11). Cases from multiple pregnancies were 1.62 times more likely to result in mortality than singletons (95% CI, 1.13–2.32). Cases with structural and chromosomal anomalies were at quadrupled risks of mortality (HR=4.22; 95% CI, 3.38–5.27; *P*<0.001 and HR=4.11; 95% CI, 3.47–4.87; *P*<0.001, respectively).

Year of birth (*P*<0.001), gestational age at birth (*P*<0.001), birth weight (*P*=0.001), CHD severity (*P*<0.001), and extracardiac anomalies (*P*<0.001) remained significant in the multivariable model, although the HRs were all attenuated compared with univariable analyses (Table 1). Plurality was no longer significant (*P*=0.22), with the effect changing direction (adjusted HR=0.78; 95% CI, 0.53–1.15). The small increased risk of mortality associated with increasing maternal age was similar, but no longer statistically significant (*P*=0.10).

### Five‐Year Conditional Survival Estimates

Most children with CHD were born at term with average birth weight (53.7%, 54.7%, 52.1%, and 56.6%, respectively for composite CHD, severity I, II, II, and unclassified CHD) and only very few were both low birth weight and very preterm (0.5%, 0.4%, 0.1%, 0.7%, and 0.1%, respectively, for any CHD, severity I, II, III, and unclassified CHD).

Five‐year survival for a child with isolated CHD, born at term in 2003 with average birth weight, was estimated as 96.3% (95% CI, 95.6–97.0). This dropped to 83.1% (95% CI, 78.5–87.8) for very preterm births and 93.4% (95% CI, 91.9–95.0) for moderately preterm births, but increased to 97.3% (95% CI, 95.8–98.7) for post‐term births. Five‐year survival for a child with isolated CHD, born at term in 2003 with low birth weight, was estimated as 95.2% (95% CI, 94.2–96.3) and with high birth weight was estimated as 97.2% (95% CI, 96.4–98.0). Children with low birth weight born very preterm had the worst prognosis (survival=78.8%; 95% CI, 72.8–84.7) and children with high birth weight born post‐term had the best prognosis (survival=97.9%; 95% CI, 96.8–99.1; Table 2).

**Table 2:** 5‐Year Survival Estimates by CHD Subtypes

CHD Subtype	Deaths/N	Kaplan–Meier Survival Estimate for Isolated CHD Born 1985–2003	Conditional Survival Estimates, for Isolated CHD Born 2003^a^					
			Term, Average Birth Weight	Preterm, Average Birth Weight	Term, Low Birth Weight	Preterm, Low Birth Weight	Term, High Birth Weight	Preterm, High Birth Weight
Severe I	108/163	34.7 (27.0–42.5)	58.8 (42.8–74.7)	38.4 (15.4–61.4)	53.5 (33.0–73.9)	32.5 (7.4–57.5)	59.7 (38.3–81.2)	39.5 (13.0–66.1)
Hypoplastic left heart	76/79	≤5.5 (1.8–12.4)^b^	···	···	···	···	···	···
Single ventricle	4/11	77.8 (36.5–93.9)	···	···	···	···	···	···
Hypoplastic right heart	4/12	63.6 (29.7–85.4)	···	···	···	···	···	···
Tricuspid atresia	16/34	63.0 (42.1–78.1)	···	···	···	···	···	···
Ebstein anomaly	8/27	70.8 (48.4–84.9)	···	···	···	···	···	···
Severity II	385/1579	81.0 (78.7–83.1)	89.2 (86.5–91.8)	79.3 (73.5–85.0)	87.9 (84.6–91.2)	76.9 (70.3–83.6)	90.8 (87.6–94.0)	82.2 (76.1–88.3)
Pulmonary valve atresia	28/66	61.6 (47.4–73.0)	61.2 (35.3–87.1)	63.0 (18.7–100)	38.6 (3.8–73.4)	40.9 (−13.3–95.1)	67.7 (37.7–97.8)	69.3 (34.7–100)
Interrupted aortic arch	13/33	52.6 (28.7–71.9)	···	···	···	···	···	···
Common arterial trunk	35/52	36.1 (21.0–51.4)	77.2 (55.4–99.0)	54.5 (14.2–94.7)	74.8 (53.6–96.0)	50.6 (9.7–91.5)	92.9 (81.4–100)	84.2 (61.1–100)
Aortic valve atresia/stenosis	30/247	89.4 (84.6–92.8)	94.6 (89.4–99.8)	91.5 (81.6–100)	93.3 (86.4–100.0)	89.6 (76.7–100)	97.5 (93.3–100)	96.0 (89.3–100)
Atrioventricular septal defect	90/264	76.6 (67.4–83.5)	89.2 (83.0–95.4)	76.0 (61.5–90.5)	82.4 (72.1–92.7)	62.8 (41.8–83.7)	93.6 (87.4–99.8)	85.3 (71.5–99.1)
Tetralogy of fallot	61/271	85.3 (79.5–89.6)	90.6 (84.6–96.5)	86.2 (76.0–96.4)	89.3 (82.2–96.4)	84.4 (72.9–95.8)	90.3 (81.8–98.9)	85.9 (72.4–99.3)
Total anomalous pulmonary venous return	19/64	72.7 (58.9–82.6)	···	···	···	···	···	···
Transposition of the great vessels	50/222	78.7 (72.4–83.7)	93.1 (88.3–98.0)	74.7 (56.7–92.6)	92.5 (86.4–98.6)	72.5 (51.1–93.9)	94.2 (88.9–99.5)	78.2 (61.8–94.7)
Coarctation of the aorta	46/258	85.2 (79.7–89.3)	86.3 (77.4–95.1)	72.6 (52.1–93.1)	90.2 (82.4–98)	79.9 (62.0–97.8)	82.8 (69.9–95.7)	66.5 (41.6–91.5)
Double outlet right ventricle	9/22	64.3 (34.3–83.3)	···	···	···	···	···	···
Severity III	118/3032	98.8 (98.3–99.1)	99.3 (99.0–99.6)	97.8 (96.6–98.9)	99.0 (98.6–99.5)	96.9 (95.2–98.5)	99.6 (99.4–99.9)	98.8 (97.9–99.7)
Pulmonary valve stenosis	10/428	98.4 (96.5–99.3)	···	···	···	···	···	···
Atrial septal defect	19/422	98.2 (96.1–99.2)	···	···	···	···	···	···
VSD	88/2182	98.9 (98.3–99.3)	99.3 (98.9–99.7)	97.3 (95.7–98.9)	99.2 (98.6–99.7)	96.7 (94.6–98.8)	99.7 (99.4–99.9)	98.6 (97.5–99.8)
Mitral valve anomalies	3/80	97.3 (89.8–99.3)	···	···	···	···	···	···
Unclassified severity	41/296	93.0 (88.5–95.8)	98.1 (96.3–99.9)	93.8 (87.8–99.8)	95.9 (91.9–100.0)	86.9 (74.6–99.2)	97.7 (95.3–100)	92.5 (84.7–100)
Patent ductus arteriosus	6/39	92.5 (87.9–95.4)	···	···	···	···	···	···

**···** indicates too few cases or deaths to produce covariate‐adjusted survival estimates; CHD, congenital heart disease; VSD, ventricular septal defect.

a Conditional survival estimates presented only where there were ≥5 deaths per variable (ie, 25 deaths).

b Six‐month survival as too few cases left at risk at age 5 years.

Five‐year survival for a child with isolated CHD, born at term in 2003 with average birth weight, was estimated as 58.8% (95% CI, 42.8–74.7) for a severity I CHD, 89.2% (95% CI, 86.5–91.8) for severity II, 99.3% (95% CI, 99–99.6) for severity III, and 98.1% (95% CI, 96.3–99.9) for unclassified. Survival for selected CHD subtypes conditional on birth weight and gestational age are shown in Table 2.

## Discussion

Year of birth, gestational age at birth, birth weight, and extracardiac anomalies were independently associated with mortality in individuals with CHD. Most children (53.7%) with isolated CHD were born at term with average birth weight. An estimated 96.3% of isolated cases born at term in 2003, with average birth weight, were alive at age 5 years. This ranged from 58.8% for children with the most severe CHD subtypes to 99.3% for the least severe. Five‐year survival was most optimistic (97.9%) for children born with high birth weight post‐term and was least optimistic (78.8%) for children born very preterm with a low birth weight.

This study's main strength is the use of data from a high‐quality, population‐based register, which is notified from multiple sources to maximize ascertainment. The NorCAS is annually cross‐validated with a pediatric cardiac database at the local tertiary center. Complex cases are reviewed by pediatric pathologists and clinical geneticists, and, where relevant, diagnoses are confirmed by postmortem. NorCAS cases may be diagnosed at any age up to 16 years (12 after 2001), meaning that difficult and late diagnoses are included. However, cases born to mothers resident in the North of England that move out of the UK before diagnosis may not be picked up by the NorCAS. Given that the North of England is a very stable population with little migration, this is likely to apply to a very few cases.^20^ Only 23 cases (0.5%) were untraced, virtually eliminating any potential bias from loss to follow‐up.

We uniquely present adjusted 5‐year survival estimates, overall, by CHD severity and by selected CHD subtypes. Existing studies typically report survival for all CHD combined, not accounting for modifying factors such as extracardiac anomalies or preterm birth. We show that these factors substantially influence survival (eg, 5‐year survival for common arterial trunk falls from 77.2% in term births with average birth weight to 50.6% for preterm births with low birth weight). Adjusted survival estimates are important for health and social care planning. For prenatal counselling, they can provide best‐ and worst‐case scenarios, depending on the final gestational age and birth weight. For postnatal diagnoses, they can provide parents with more‐accurate predictions based on their baby's birth weight and gestational age.

Although one of the largest studies to examine the influences of CHD survival, we still lacked power to assess uncommon features (eg, high birth weight or post‐term birth) or factors with modest effect sizes (eg, socioeconomic deprivation). Nonsignificant associations should therefore be interpreted with care.

Simulation studies suggest that survival analysis requires a minimum of 5 to 10 events per variable for adequate statistical power.^21^, ^22^, ^23^ Our adjusted survival estimates for individual subtypes and severity categories were derived from models containing 4 dummy categorical variables and 1 continuous variable. To accurately report survival, we therefore required at least 20 deaths for each CHD subtype, which meant several subtypes could not be examined individually.

Ethnicity and parity have previously been associated with CHD mortality.^6^, ^7^, ^8^, ^9^ Data notified to the NorCAS are collected routinely in clinical settings, and these variables are poorly recorded. Surgical and medical interventions are also not recorded on the NorCAS, but likely impact survival. In particular, for cases of hypoplastic left heart, survival may be improved with palliative surgery, though many parents opt for comfort care.^24^ Moreover, younger age at surgical intervention appears to improve survival in children with CHD.^25^, ^26^, ^27^, ^28^, ^29^ The NorCAS does not hold information on morbidities such as sepsis or pulmonary hypertension, which are more prevalent in children with CHD and increase the risk of mortality.^29^, ^30^, ^31^

Without information on cause of death, we cannot confirm whether a cardiac event was the cause of death. Mortality among cases with extracardiac anomalies may result from the coincident anomaly, rather than the CHD. However, for severe subtypes, the contribution of any additional anomalies is diminished by the CHD lethality. To provide the most widely relevant figures, we presented our adjusted survival estimates for isolated CHD only.

We found that CHD survival improved over time during 1985–2003. We therefore fixed our survival estimates to the latest year of study. Survival prospects are likely to have improved since 2003, and this should be considered when interpreting our estimates. Assuming trends increased at the same rate until 2016, survival for cases born at term, with average birth weight and isolated CHD, would have increased form 96.3% to 98.7%. Similarly, survival for cases born very preterm with low birth weight would have increased from 78.8% in 2003 to 91.9% in 2016. Survival for cases born post‐term with high birth weight would have increased from 97.9% in 2003 to 99.3% in 2016.

Our Kaplan–Meier survival estimate of 87.8% at age 5 years is comparable to the pooled estimate of 85% reported in our recent systematic review^3^ (after excluding Tennant et al, which used overlapping data^18^).

In our study, cases with extracardiac anomalies experienced ≈4‐fold increased risk of mortality. Knowles et al and Olsen et al similarly reported that children with extracardiac anomalies were at increased risk of mortality, but with smaller effect sizes.^11^, ^29^ These discrepancies may result from Knowles et al excluding cases with Down syndrome and Olsen et al having a different case mix, with just half the proportion of ventricular septal defects than our study (23% versus 43%).

We found that CHD survival improved over time, reflecting the findings of several other population‐based studies.^5^, ^8^, ^11^, ^32^ This improvement is likely explained by many factors, including the development of several surgical interventions. For example, the Fontan operation and the Norwood surgery were introduced in the UK in 1975 and 1993, respectively, to enable palliative treatment for single ventricle, hypoplastic left heart, and tricuspid atresia.^33^ Similarly, the arterial switch operation was introduced to the UK in 1984, replacing the atrial switch operations. Improving expertise as well as general improvements in neonatal care (which enable survival until surgical intervention) are likely to have contributed to the ongoing improvements in survival. For example, prostaglandin was introduced to the UK in 1978, helping patients to remain stable before surgical intervention.^34^

We found that greater gestational age at birth was associated with improved survival. Knowles et al also reported an increased risk of mortality in preterm compared with term cases (HR=1.43).^29^ The slightly smaller effect size may result from Knowles et al examining only “serious CHD.” Fixler et al similarly reported an increased risk of mortality in very preterm cases (HR=2.80) and moderately preterm cases (HR=1.69), but only included CHD subtypes with single‐ventricle physiology.^4^ We found that increased standardized birth weight was associated with improved survival. Wang et al and Oster et al similarly reported that increased birth weight improved survival.^8^, ^32^ Cardiac operative mortality has been shown to increase with lower birth weight and lower gestational age at birth. Furthermore, among children with CHD, low gestational age at birth poses an increased risk of necrotizing entercolitis.^35^ Of course, in individuals without CHD, the risk of mortality increases as gestational age and birth weight decreases.^36^, ^37^ However, our increased risk of mortality associated with moderately preterm birth in individuals with CHD (HR=2.09) exceeds that reported by Crump et al in the general population (HR=1.80 for babies born 28–33 weeks and HR=1.52 for babies born 34–36 weeks versus 37–42 weeks).^36^

We found some evidence of an association between maternal age at delivery and mortality. Wang et al and Oster et al reported significantly decreased mortality in cases born to mothers respectively aged >35 years compared with 30 to 34 years (HR=0.88)^8^ and aged ≥30 years compared with <30 years (HR=0.77),^32^ respectively. Other population‐based studies have reported no significant association between CHD survival and maternal age in cases with single‐ventricle physiology^4^, ^6^ and atrioventricular septal defect, but showed lower survival in children born to older mothers. Potentially the association with maternal age is confounded by other factors, such as CHD subtype, socioeconomic deprivation, and gestational age at birth, which all varied by maternal age in our data.

We found lower survival in cases from multiple pregnancies. However, after accounting for other variables, this association was no longer significant. In our data, cases from multiple births were 10 times more likely to be preterm, 1.5 times more likely to have low birth weight, and 2.5 times more likely to have structural extracardiac anomalies. Therefore, although twins on average have poorer prognoses, a term, average‐weight twin with isolated CHD should have the same survival prospects as an equivalent singleton.

We found poorer survival of children with CHD born in the most compared with the least deprived areas in the North of England. Miller et al did not find a significant association between socioeconomic position and atrioventricular septal defect survival in the USA; however, survival decreased linearly with decreasing level of deprivation.^10^ Area‐based deprivation is a complex and multifaceted exposure, with many domains and correlates, only some of which may be related to CHD survival. Detecting such an association may require a larger data set with greater power or more detail on the individual features of the exposure. While the impact may be small for the individual, it may still be an important determinant at the population level.

## Conclusion

Twenty‐year survival associated with CHD was 85.2%. Year of birth, gestational age at birth, standardized birth weight, and the presence of extracardiac anomalies were associated with mortality in individuals with CHD. This information is important for health and social care planning.

## Sources of Funding

This work was supported by a British Heart Foundation studentship (FS/12/23/29511 to Best). NorCAS is currently funded by Public Health England.

## Disclosures

None.

## References

[1] EUROCAT . UK congenital anomaly register prevalence tables. 2013 Available at: http://www.eurocat-network.eu/accessprevalencedata/prevalencetables. Accessed April 24, 2017.

[2] Wren C , O'Sullivan JJ . Survival with congenital heart disease and need for follow up in adult life. Heart. 2001;85:438–443. 11250973 10.1136/heart.85.4.438 PMC1729699

[3] Best KE , Rankin J . Long‐term survival of individuals born with congenital heart disease: a systematic review and meta‐analysis. J Am Heart Assoc. 2016;5:e002846 https://doi.org/10.1161/jaha.115.002846. 27312802 10.1161/JAHA.115.002846 PMC4937249

[4] Fixler DE , Nembhard WN , Salemi JL , Ethen MK , Canfield MA . Mortality in first 5 years in infants with functional single ventricle born in Texas, 1996 to 2003. Circulation. 2010;121:644–650. 20100974 10.1161/CIRCULATIONAHA.109.881904

[5] Garne E . Congenital heart defects—occurrence, surgery and prognosis in a Danish County. Scand Cardiovasc J. 2004;38:357–362. 15804803 10.1080/14017430410024379

[6] Idorn L , Olsen M , Jensen AS , Juul K , Reimers JI , Sorensen K , Johnsen SP , Sondergaard L . Univentricular hearts in Denmark 1977 to 2009: incidence and survival. Int J Cardiol. 2013;167:1311–1316. 22521378 10.1016/j.ijcard.2012.03.182

[7] Nembhard WN , Salemi JL , Ethen MK , Fixler DE , Dimaggio A , Canfield MA . Racial/ethnic disparities in risk of early childhood mortality among children with congenital heart defects. Pediatrics. 2011;127:e1128–e1138. 21502234 10.1542/peds.2010-2702

[8] Wang Y , Hu J , Druschel CM , Kirby RS . Twenty‐five‐year survival of children with birth defects in New York State: a population‐based study. Birth Defects Res A Clin Mol Teratol. 2011;91:995–1003. 21960515 10.1002/bdra.22858

[9] Wang Y , Liu G , Druschel CM , Kirby RS . Maternal race/ethnicity and survival experience of children with congenital heart disease. J Pediatr. 2013;163:1437–1442.e1431–1432. 23932315 10.1016/j.jpeds.2013.06.084

[10] Miller A , Siffel C , Lu C , Riehle‐Colarusso T , Frías JL , Correa A . Long‐term survival of infants with atrioventricular septal defects. J Pediatr. 2010;156:994–1000. 20227717 10.1016/j.jpeds.2009.12.013

[11] Olsen M , Christensen TD , Pedersen L , Johnsen SP , Hjortdal VE . Late mortality among Danish patients with congenital heart defect. Am J Cardiol. 2010;106:1322–1326. 21029832 10.1016/j.amjcard.2010.06.062

[12] EUROCAT . EUROCAT guide 1.4. 2014 Available at: http://www.eurocat-network.eu/aboutus/datacollection/guidelinesforregistration/guide1_4. Accessed April 24, 2017.

[13] Gardosi J , Mongelli M , Wilcox M , Chang A . An adjustable fetal weight standard. Ultrasound Obstet Gynecol. 1995;6:168–174. 8521065 10.1046/j.1469-0705.1995.06030168.x

[14] Tin W , Wariyar UK , Hey EN . Selection biases invalidate current low birthweight weight‐for‐gestation standards. BJOG. 1997;104:180–185. 10.1111/j.1471-0528.1997.tb11041.x 9070135

[15] Noble M , Wright G , Smith G , Dibben C . Measuring multiple deprivation at the small‐area level. Environ Plan A. 2006;38:169.

[16] Department for Communities & Local Government . English indices of deprivation 2010. 2010.

[17] Khoshnood B , Loane M , Garne E , Addor MC , Arriola L , Bakker M , Barisic I , Bianca S , Boyd P , Calzolari E , Doray B , Draper E , Gatt M , Haeusler M , Melve KK , Latos‐Bielenska A , McDonnell B , Mullaney C , Nelen V , O'Mahony M , Pierini A , Queisser‐Luft A , Randrianaivo H , Rankin J , Rissmann A , Salvador J , Tucker D , Verellen‐Dumoulin C , Wellesley D , Zymak‐Zakutnya N , Dolk H . Recent Decrease in the Prevalence of Congenital Heart Defects in Europe. J Pediatr. 2012;162:108–113. 22835879 10.1016/j.jpeds.2012.06.035

[18] Tennant PW , Pearce MS , Bythell M , Rankin J . 20‐year survival of children born with congenital anomalies: a population‐based study. Lancet. 2010;375:649–656. 20092884 10.1016/S0140-6736(09)61922-X

[19] Royston P , Lambert P . Flexible Paramteric Survival Analyses Using Stata: Beyond the Cox Model. Texas: Stata Press; 2011.

[20] Hodgson S , Shirley M , Bythell M , Rankin J . Residential mobility during pregnancy in the north of England. BMC Pregnancy Childbirth. 2009;9:52. 19912662 10.1186/1471-2393-9-52 PMC2784435

[21] Concato J , Peduzzi P , Holford TR , Feinstein AR . Importance of events per independent variable in proportional hazards analysis I. Background, goals, and general strategy. J Clin Epidemiol. 1995;48:1495–1501. 8543963 10.1016/0895-4356(95)00510-2

[22] Peduzzi P , Concato J , Feinstein AR , Holford TR . Importance of events per independent variable in proportional hazards regression analysis. II. Accuracy and precision of regression estimates. J Clin Epidemiol. 1995;48:1503–1510. 8543964 10.1016/0895-4356(95)00048-8

[23] Vittinghoff E , McCulloch CE . Relaxing the rule of ten events per variable in logistic and Cox regression. Am J Epidemiol. 2007;165:710–718. 17182981 10.1093/aje/kwk052

[24] Fruitman DS . Hypoplastic left heart syndrome: prognosis and management options. Paediatr Child Health. 2000;5:219. 20177524 10.1093/pch/5.4.219 PMC2817797

[25] Mahle WT , Spray TL , Wernovsky G , Gaynor JW , Clark BJ . Survival after reconstructive surgery for hypoplastic left heart syndrome a 15‐year experience from a single institution. Circulation. 2000;102:III‐136–III‐141. 10.1161/01.cir.102.suppl_3.iii-136 11082376

[26] Murphy JG , Gersh BJ , McGoon MD , Mair DD , Porter CJ , Ilstrup DM , McGoon DC , Puga FJ , Kirklin JW , Danielson GK . Long‐term outcome after surgical repair of isolated atrial septal defect: follow‐up at 27 to 32 years. N Engl J Med. 1990;323:1645–1650. 2233961 10.1056/NEJM199012133232401

[27] Pigula FA , Khalil PN , Mayer JE , Pedro J , Jonas RA . Repair of tetralogy of fallot in neonates and young infants. Circulation. 1999;100:II‐157–II‐161. 10.1161/01.cir.100.suppl_2.ii-157 10567296

[28] Boening A , Scheewe J , Heine K , Hedderich J , Regensburger D , Kramer HH , Cremer J . Long‐term results after surgical correction of atrioventricular septal defects. Eur J Cardiothorac Surg. 2002;22:167–173. 12142181 10.1016/s1010-7940(02)00272-5

[29] Knowles RL , Bull C , Wren C , Wade A , Goldstein H , Dezateux C . Modelling survival and mortality risk to 15 years of age for a national cohort of children with serious congenital heart defects diagnosed in infancy. PLoS One. 2014;9:e106806. 25207942 10.1371/journal.pone.0106806 PMC4160226

[30] Ascher SB , Smith PB , Clark RH , Cohen‐Wolkowiez M , Li JS , Watt K , Benjamin DK . Sepsis in young infants with congenital heart disease. Early Hum Dev. 2012;88:S92–S97. 22633525 10.1016/S0378-3782(12)70025-7 PMC3513769

[31] van Loon RLE , Roofthooft MTR , Hillege HL , ten Harkel ADJ , van Osch‐Gevers M , Delhaas T , Kapusta L , Strengers JLM , Rammeloo L , Clur S‐AB , Mulder BJM , Berger RMF . Pediatric pulmonary hypertension in the Netherlands Clinical Perspective. Epidemiology and characterization during the period 1991 to 2005. Circulation. 2011;124:1755–1764. 21947294 10.1161/CIRCULATIONAHA.110.969584

[32] Oster ME , Lee KA , Honein MA , Riehle‐Colarusso T , Shin M , Correa A . Temporal trends in survival among infants with critical congenital heart defects. Pediatrics. 2013;131:e1502–e1508. 23610203 10.1542/peds.2012-3435 PMC4471949

[33] Knowles RL , Bull C , Wren C , Dezateux C . Mortality with congenital heart defects in England and Wales, 1959–2009: exploring technological change through period and birth cohort analysis. Arch Dis Child. 2012;97:861–865. 22753769 10.1136/archdischild-2012-301662

[34] Olley PM , Coceani F , Bodach E . E‐type prostaglandins: a new emergency therapy for certain cyanotic congenital heart malformations. Circulation. 1976;53:728–731. 56243 10.1161/01.cir.53.4.728

[35] McElhinney DB , Hedrick HL , Bush DM , Pereira GR , Stafford PW , Gaynor JW , Spray TL , Wernovsky G . Necrotizing enterocolitis in neonates with congenital heart disease: risk factors and outcomes. Pediatrics. 2000;106:1080–1087. 11061778 10.1542/peds.106.5.1080

[36] Crump C , Sundquist K , Sundquist J , Winkleby MA . Gestational age at birth and mortality in young adulthood. JAMA. 2011;306:1233–1240. 21934056 10.1001/jama.2011.1331

[37] Risnes KR , Vatten LJ , Baker JL , Jameson K , Sovio U , Kajantie E , Osler M , Morley R , Jokela M , Painter RC , Sundh V , Jacobsen GW , Eriksson JG , Sorensen TI , Bracken MB . Birthweight and mortality in adulthood: a systematic review and meta‐analysis. Int J Epidemiol. 2011;40:647–661. 21324938 10.1093/ije/dyq267

